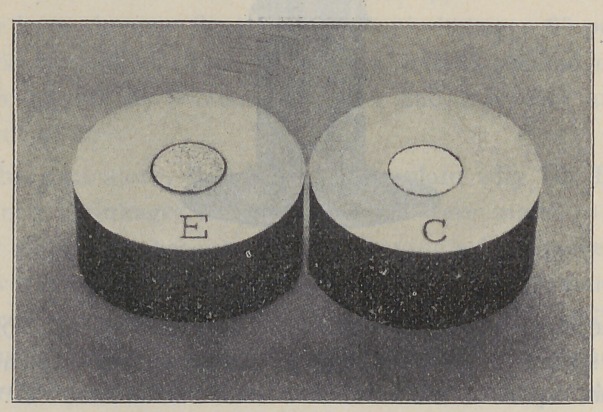# Do Modern Alloys Shrink?

**Published:** 1905-06-15

**Authors:** M. L. Ward

**Affiliations:** Ann Arbor, Mich.


### DO MODERN ALLOYS SHRINK?

BY M. L. WARD, D.D.S., ANN ARBOR, MICH.

What protection has the practitioner from unreliable articles about amalgams in our periodic literature? One writer presents an article advocating the use of modern alloys in preference to the older makes, and presumes to present with it scientific reasons for the use of them. Some one else presents an article the very next month, which not only reflects upon the careful scientific work of Dr. Black, but lauds the older alloys that were made in a period in which nothing definite was known about them except by clinical experimentation.

Even the most casual observer who has given the subject *any* consideration must be impressed with the injustice of such contradictory articles which serve only to confuse the general practitioner. Scarcely a month passes but the truth of these assertions are brought forcibly to mind. The journals are full of such instances, and promise to continue so unless some one who has the time, energy and facilities for carrying on such work, takes the matter in hand and furnishes criticism enough to make the authors of such articles a little more conservative in making damaging dogmatic comparisons.

A perusal of the literature in connection with this subject of amalgam alloys, however inconsequential, reveals a tendency on the part of the older men in the profession to give undue credit to such men as Townsend, Flagg, Fletcher and others, and ignore the only reliable scientific work we have, viz., that of Dr. Black.

This tendency is more pronounced in some instances than others and seems dependent largely upon the locality of the writers, their associations, or the men from whom they received their early instructions, but the ideas can be traced all through our periodic literature.

Since my work on this subject began, a very large percent of the alloys used throughout this section of the country have changed from those having a low to those having a high percentage of silver, and are made to coincide with Dr. Black's findings. I refer to this with satisfaction and feel called upon to volunteer a defense against the mistaken findings of an article by W. St. George Elliott, M. D., D. D. S., of New York City, as published in the April, 1905, *Dental Cosmos*.

He states that 20th Century, Fellowship, True Dent and Acme alloys shrink, and that Townsend's alloy gives good results, according to his methods of determining shrinkage. He also states that his experiments show that silver shrinks in hardening, that he knows of no amalgam that is quite reliable, etc.

I sincerely hope, that should any one desire to change from 20th Century, Fellowship, True Dent or Acme alloys, that they will not turn to Townsend's alloy, which he says makes an excellent showing, because it certainly does *not* deserve supremacy.

I am not willing to concede that because he uses the specific gravity method of determining shrinkage and expansion that he obtains more accurate results than those who use the micrometer, nor am I willing to concede that the first four named alloys are shrinking alloys under all circumstances. I *am* willing to admit that a micrometer gives only linear measurements, and that excessive expansions may be obtained by converting lateral expansion into a flow of the metal upward by making the tests in sufficiently resistant receptacles, and, further, that the specific gravity method of determining shrinkage and expansion is the one generally adopted by physicists. However, I am not ready to admit that the micrometer is not the proper device for measuring the shrinkage and expansion of amalgams.

Dr. Elliott has evidently overlooked a very important feature in this work, viz., that the modern alloys are high percentage silver alloys, and the Townsend's is a very low one. An analysis made within the last ten days of Townsend's alloy showed the presence of:

42.02 percent of silver.
57.56 percent of tin.
.42 percent of copper.

The Twentieth Century, Fellowship, True Dent and Acme have shown a fluctuating formula between 65 percent and 68 percent of silver, 26 percent and 28 percent of tin, 3 percent and 4½ percent of copper and 1 percent and 2½ percent of zinc.

This fact alone, is sufficient to explain why Dr. Elliott obtained shrinkages from alloys that were made to show slight expansion, because the ones having a high percentage of silver have a great affinity for mercury, a greater tendency towards crystallization, and break down slowly, while the Townsend's has a less affinity for mercury, a less tendency toward crystallization and breaks down easily. As we progress it will become apparent that shrinkage may be a change in volume or a change in dimensions and that the two are distinctly separate in this work.

For the benefit of the puzzled practitioner we will review the specific gravity method of determining shrinkage and expansion and let each judge for himself whether it is a desirable method to pursue in this connection.

When a body is immersed in any fluid it apparently loses weight because it is supported by the fluid or is subjected to an upward pressure equal to the weight of the fluid displaced.

The upward pressure on the immersed body is the same as that on the fluid which it displaces. Let a filling of amalgam be suspended from one arm of a balance in a vessel of water, such as is shown in figure No. 1, and it will be seen at once that the opposite lateral sides "A-B" will be equally pressed in opposite directions.

*Fig.* 1.

The same will be true on all sides of the filling. On the upper surface of the filling at "D" there will be a downward pressure equal to the column of water with base "D" and height "N-D." On the bottom there will be an upward pressure equal to the weight of the column of water whose base is "C" and height "N-C." The resultant upward pressure on the filling is the difference of the pressures on the bottom and top of the filling, and this difference is the weight of the cylinder of water of the same dimensions as the cylinder of amalgam.

From this it may be seen that if the amalgam shrinks it will weigh more and if it expands it will weigh less.

When an amalgam filling is made and packed into a cavity, or other receptacle, the surfaces nearly always contain more mercury than the center of the filling, because it is impossible to condense the borders as much as the center and as a result the mercury is displaced and carried to the borders there to be reabsorbed and equally distributed throughout the filling as time goes on. In the cases where the mercury is not carried to the borders, as well as the ones just mentioned, the surface of the filling is usually smooth and bright when first made, but as soon as the amalgam begins to set the surface becomes porous.

The pores thus formed are seen to be of varying sizes and are formed with varying degrees of rapidity, both of which are greater with the high percentage, and less with the low percentage silver alloys.

Figure "C" shows a surface of a high percentage silver filling which was photographed within one hour after it was made. Figure "E" shows a companion filling made under exactly the same conditions but is two days old. It may be noticed that in Fig. "C" the surface is bright and smooth while "E," which is two days old, has become porous upon the surface.

The actual dimensions and actual weight of this filling have not changed one iota though it can be readily seen that if this filling had been suspended in water when it was first made, as it appears in Fig. "C," it would have displaced a greater volume of water than it would two days later after it became porous on the surface and appeared as shown in Fig. "E." These pores formed on the surface would be regarded by physicists as shrinkage, while a micrometer measurement taken by making a contact on one or both ends would show absolutely no change.

It would be possible to get an actual increase in the dimensions of the filling and still have a specific gravity test show shrinkage.

This is undoubtedly the case with Dr. Elliott's test, because these are some of our best alloys and are not showing shrinkage when tested with a micrometer.

From this it may be seen that we, who are using a micrometer, regard shrinkage as decrease in dimensions, which is decrease in magnitude measured along a diameter, while Dr. Elliott, who uses the specific gravity method, regards shrinkage as decrease in volume which includes the pores or interstices formed along such a diameter.

The volume of a body, then, may be "real" or "apparent," the "real" volume being the space occupied by the actual substance of which the body is composed, after making allowance for the pores or interstices that may be present, while the "apparent" volume is the space included within an imaginary surface which just takes in the body, interstices and all.

From this it may be seen that it is the "real" volume that Dr. Elliott measured by his specific gravity method, and that the "apparent" volume or "dimensions" is what we believe should be measured.

The question naturally arises, which method should be pursued? It is evident that the dimensions and not the "real volume" will fracture walls of enamel, compress pulps, protrude over the borders or allow the filling to fall out, and must be measured if these defects are overcome. Of course, it is interesting to know how many and with what rapidity these pores are formed on the surfaces, but that is an entirely different matter and should not be used to reflect upon the work of scientific amalgam manufacturers who are following closely modern methods.

The cause, extent and rapidity with which porosity on the surface is formed, depends upon the affinity of the constituents of the alloy for mercury and the completeness with which the alloy is dissolved in the mercury. Silver is the constituent that determines this largely, because of its affinity for mercury and crystalline form. When we consider that the modern alloys contain from 65 percent to 68 percent of silver and that Townsend's contains only 42 percent, we may readily see that the affinities of the two alloys for mercury is not to be compared, and that the large percentage of tin in the Townsend's will allow this alloy to be completely dissolved with very little manipulation, regardless of its being cut much coarser.

The specific gravity method will measure change in dimensions as well as change in "real volume," but the alloys that are made as close as 1-20,000 of an inch expansion on a filling one-quarter inch high and one-quarter inch in diameter, when measured with a micrometer, are made so near to neutral that the porosity formed on their surfaces in the early stages of crystallization is greater than the actual increase in dimensions.

Dr. Elliott states that in Dr. Flagg's book he mentions silver as being the expansive agent. He neglects to mention that Dr. Black published a table ten years ago showing the action of silver in varying proportions with tin and that no one has disproved it since. Dr. Elliott says that his experiments show that silver is a shrinking component. The fact is that expansion from silver and mercury may be seen with the naked eye, not a single instrument is necessary to detect it. It must be remembered, however, that silver unites with mercury slowly at ordinary temperatures, and that the finer the state of division the more rapid will be the union. Reduced silver will unite with mercury and produce an expansion within three minutes, while the ordinary filings of silver would require weeks or months to harden and produce much of an expansion if kept at room temperatures, but the expansion in either case can be seen with the naked eye. The following table was published in the *Cosmos* Vol. 36, page 982, and shows results of Dr. Black's work upon the shrinkage and expansion of silver and tin.

EXHIBIT OF UNMODIFIED SILVER—TIN ALLOYS.

| Formulae | | How Prepared | Percent of Mercury | Shrinkage | Expansion | Flow | Crushing Stress |
|---|---|---|---|---|---|---|---|
| Silver | Tin | | | | | | |
| 40 | 60 | Fresh Cut | 45.78 | 6 | 7 | 40.15 | 178 |
| 40 | 60 | Annealed | 34.14 | 9 | 3 | 44.60 | 186 |
| 45 | 55 | Fresh Cut | 49.52 | 4 | 8 | 25.46 | 188 |
| 45 | 55 | Annealed | 32.13 | 11 | 1 | 28.57 | 222 |
| 50 | 50 | Fresh Cut | 51.18 | 2 | 2 | 22.16 | 232 |
| 50 | 50 | Annealed | 37.58 | 17 | 1 | 21.03 | 245 |
| 55 | 45 | Fresh Cut | 51.62 | 2 | 2 | 19.66 | 245 |
| 55 | 45 | Annealed | 40.11 | 18 | 0 | 17.53 | 276 |
| 60 | 40 | Fresh Cut | 52.00 | 1 | 0 | 9.06 | 239 |
| 60 | 40 | Annealed | 39.80 | 17 | 0 | 14.10 | 297 |
| 65 | 35 | Fresh Cut | 52.00 | 0 | 1 | 3.67 | 290 |
| 65 | 35 | Annealed | 33.00 | 10 | 0 | 5.00 | 335 |
| 70 | 30 | Fresh Cut | 55.00 | 0 | 14 | 3.45 | 216 |
| 70 | 30 | Annealed | 40.00 | 7 | 0 | 4.67 | 375 |
| 72.5 | 27.5 | Fresh Cut | 55.00 | 0 | 42 | 3.92 | 275 |
| 72.5 | 27.5 | Annealed | 45.00 | 3 | 0 | 3.76 | 362 |
| 75 | 25 | Fresh Cut | 55.00 | 0 | 60 | 5.64 | 258 |
| 75 | 25 | Annealed | 50.00 | 0 | 6 | 5.40 | 300 |

Dr. Elliott states that he generally confines his observations to forty-eight hours. Any one who has studied the changes that take place in the setting of plasters, cements or alloys, knows very well that these or any of the associated processes of crystallization are not completed in forty-eight hours if they are kept at ordinary temperatures. They require weeks, months and sometimes years to be completed. Any one can easily determine that an alloy is only partially dissolved in the mercury even if it has been worked thoroughly.

The alloys containing large percentage of silver are only attacked upon the surface of each particle and as a result a greater or less portion of each is left undissolved, depending upon the cut and amount of manipulation it receives during amalgamation. With this understood how can we expect the mercury to be removed so completely or distributed so equally that no further action takes place after forty-eight hours.

It has been my experience that ten days is as short a time as any test should be kept under observation, and that at the end of six months changes are often found to be going on.

Dr. Elliott also states alloys are most sensitive to the presence of foreign matter and that for this reason he cannot give any hope that an alloy will be produced in the near future that is quite reliable. I would ask him to explain what foreign matter he expects to be picked up during amalgamation, and its effect upon the alloys of silver, tin, copper, zinc and mercury. If he has a good alloy he has one that is made from refined materials, and the trial formula is balanced to each batch of materials from which the alloy is made, and if he has good mercury it is re-distilled so that the only chance for him to pick up foreign material is during amalgamation.

He states that "we only get a mechanical mixture" when we melt and cast alloys. I would be much interested in any evidence that he might produce to prove that alloys of silver, tin, copper and zinc are mechanical mixtures. He further states that an elaborate apparatus is not necessary in conducting these experiments; if he will but give us his work in detail proving his assertions, we think we can demonstrate the value of a laboratory well fitted with accurate apparatus for the successful solution of this problem.

## Figures and Tables

**Fig. 1. f1:**
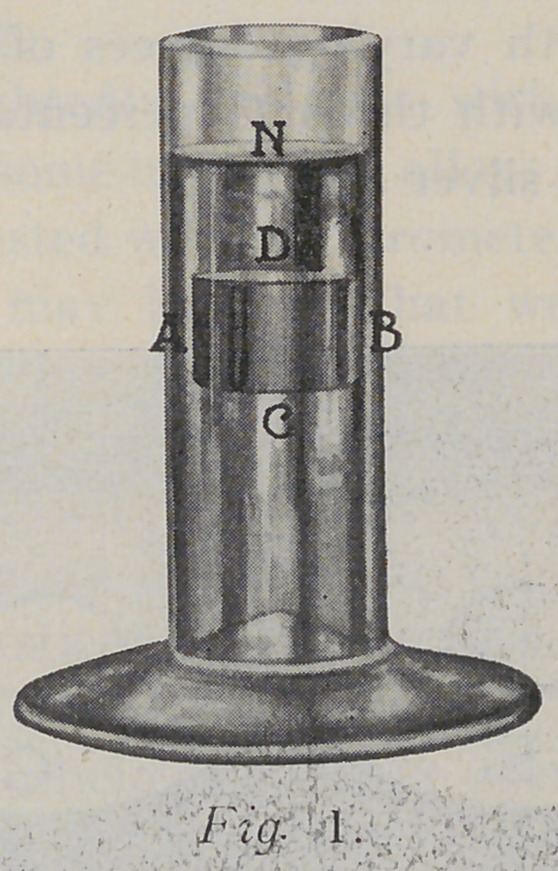


**Figure f2:**